# Supplementary material for: Empirical methods for controlling false positives and estimating confidence in ChIP-Seq peaks
Source: BMC Bioinformatics. 2008 Dec 5;9:523. doi: 10.1186/1471-2105-9-523 (PMC2628906; doi:10.1186/1471-2105-9-523)
Supplement: Additional File 1 — A variety of html documents from the USeq web site detailing the available applications, their best usage, output file type descriptions, command line menus, etc. [file 1471-2105-9-523-S1.zip › USeqUserGuides/JavaDoc.zip!/JavaDoc/trans/anno/BindingRegion.html]

BindingRegion


|  |  |  |
| --- | --- | --- |
| **Overview** \| **Package** \| **Class** \| **Use** \| **Tree** \| **Deprecated** \| **Index** \| **Help** | |  |
| **PREV CLASS**   **NEXT CLASS** | **FRAMES**    **NO FRAMES**     **All Classes** |  |
| SUMMARY: NESTED \| FIELD \| CONSTR \| METHOD | DETAIL: FIELD \| CONSTR \| METHOD |  |


---


## trans.anno Class BindingRegion

```
java.lang.Object
  trans.anno.BindingRegion
```

**All Implemented Interfaces:**: java.lang.Comparable

---

``` public class BindingRegion extends java.lang.Object implements java.lang.Comparable ```

For holding information about a genomic interval, what genes are up, down, overlap, or contain this genomic inteval.

---

**Constructor Summary**

`BindingRegion(int rank,
double score,
java.lang.String chromosome,
int start,
int end,
int sizeNeighborhood)`

`BindingRegion(Interval interval,
int rank)`

`BindingRegion(int rank,
java.lang.String chromosome,
int start,
int end)`


| **Method Summary** | |
| --- | --- |
| `int` | `bpIntersectionSameChromosome(BindingRegion other)`             Assumes regions are on the same chromosome, and the end base is included, not interbase numbering. |
| `void` | `compare(GeneGroup geneGrp)`             Compares a binding region to a gene grp looking for where it is in relation to the binding region, 5', 3', overlap, contained. |
| `int` | `compareTo(java.lang.Object obj)` |
| `static java.lang.String` | `extractGeneGrps(java.util.ArrayList geneGrps, boolean returnSummaryLine)`             Returns summary line or name for each GeneGrp in ArrayList |
| `java.lang.String` | `getChromosome()` |
| `BindingRegion` | `getClosestBindingRegion()` |
| `java.util.ArrayList` | `getContainingGeneGrps()` |
| `int` | `getDistanceTo3PrimeGeneGrp()` |
| `int` | `getDistanceTo5PrimeGeneGrp()` |
| `int` | `getEnd()` |
| `double` | `getGcContent()` |
| `GeneGroup` | `getGeneGrp3Prime()` |
| `GeneGroup` | `getGeneGrp5Prime()` |
| `int` | `getLength()`             End-Start+1 |
| `int` | `getNeighborhood()` |
| `java.util.ArrayList` | `getNeighboringGeneGrps()` |
| `java.util.ArrayList` | `getOverlap3PrimeGeneGrps()` |
| `java.util.ArrayList` | `getOverlap5PrimeGeneGrps()` |
| `int` | `getRank()` |
| `double` | `getScore()` |
| `int` | `getStart()` |
| `int` | `intersectReturnBP(BindingRegion other)`             Returns -1 for no overlap or the # bases of intersection. |
| `int` | `intersectReturnGap(BindingRegion other)`             Returns -1 if on diff chromosomes, 0 if they overlap, or the # of bases between the two. |
| `boolean` | `intersects(BindingRegion other)` |
| `int` | `overlap(BindingRegion other)`             Returns -1 for no overlap, 0 for complete overlap, or a positive int for the # bases of overlap. |
| `void` | `setChromosome(java.lang.String chromosome)` |
| `void` | `setClosestBindingRegion(BindingRegion closestBindingRegion)` |
| `void` | `setGcContent(double gcContent)` |
| `void` | `setNeighborhood(int neighborhood)` |
| `java.lang.String` | `simpleSummaryLine()`             Returns a binding region line, tab delimited: chrom, start, end, score |
| `java.lang.String` | `simpleSummaryLineWithRank()`             Returns a binding region line, tab delimited: chrom, start, end, score |
| `java.lang.String` | `summaryLine()`             Returns tab delimited line: rank, chrom, start, end, dist to closes gene, gene name(s), # neighbors |
| `java.lang.String` | `toString()` |

**Methods inherited from class java.lang.Object**

`equals, getClass, hashCode, notify, notifyAll, wait, wait, wait`

**Constructor Detail**

### BindingRegion

```
public BindingRegion(int rank,
                     double score,
                     java.lang.String chromosome,
                     int start,
                     int end,
                     int sizeNeighborhood)
```

---


### BindingRegion

```
public BindingRegion(int rank,
                     java.lang.String chromosome,
                     int start,
                     int end)
```

---


### BindingRegion

```
public BindingRegion(Interval interval,
                     int rank)
```


**Method Detail**

### compareTo

```
public int compareTo(java.lang.Object obj)
```

:   **Specified by:**: `compareTo` in interface `java.lang.Comparable`

---


### intersects

```
public boolean intersects(BindingRegion other)
```

---


### intersectReturnGap

```
public int intersectReturnGap(BindingRegion other)
```

:   Returns -1 if on diff chromosomes, 0 if they overlap, or the # of bases between the two.

---


### bpIntersectionSameChromosome

```
public int bpIntersectionSameChromosome(BindingRegion other)
```

:   Assumes regions are on the same chromosome, and the end base is included, not interbase numbering.
    Returns 0 if the regions abut, positive ints for each base in the gap (ie 1= 1bp between an end of 12 and a start of 14),
    negative ints for each base of intersection (ie -1= 1bp overlap),
    max negative int is the length of the smaller region (ie end - start +1).

---


### intersectReturnBP

```
public int intersectReturnBP(BindingRegion other)
```

:   Returns -1 for no overlap or the # bases of intersection.

---


### overlap

```
public int overlap(BindingRegion other)
```

:   Returns -1 for no overlap, 0 for complete overlap, or a positive int for the # bases of overlap.

---


### simpleSummaryLine

```
public java.lang.String simpleSummaryLine()
```

:   Returns a binding region line, tab delimited: chrom, start, end, score

---


### simpleSummaryLineWithRank

```
public java.lang.String simpleSummaryLineWithRank()
```

:   Returns a binding region line, tab delimited: chrom, start, end, score

---


### summaryLine

```
public java.lang.String summaryLine()
```

:   Returns tab delimited line: rank, chrom, start, end, dist to closes gene, gene name(s), # neighbors

---


### toString

```
public java.lang.String toString()
```

:   **Overrides:**: `toString` in class `java.lang.Object`

---


### extractGeneGrps

```
public static java.lang.String extractGeneGrps(java.util.ArrayList geneGrps,
                                               boolean returnSummaryLine)
```

:   Returns summary line or name for each GeneGrp in ArrayList

---


### compare

```
public void compare(GeneGroup geneGrp)
```

:   Compares a binding region to a gene grp looking for where it is in relation to the binding
    region, 5', 3', overlap, contained.
    Saves all that overlap or are contained as well as the closest 5' and the closest 3'.

---


### getChromosome

```
public java.lang.String getChromosome()
```

---


### getContainingGeneGrps

```
public java.util.ArrayList getContainingGeneGrps()
```

---


### getDistanceTo3PrimeGeneGrp

```
public int getDistanceTo3PrimeGeneGrp()
```

---


### getDistanceTo5PrimeGeneGrp

```
public int getDistanceTo5PrimeGeneGrp()
```

---


### getGeneGrp3Prime

```
public GeneGroup getGeneGrp3Prime()
```

---


### getGeneGrp5Prime

```
public GeneGroup getGeneGrp5Prime()
```

---


### getOverlap3PrimeGeneGrps

```
public java.util.ArrayList getOverlap3PrimeGeneGrps()
```

---


### getOverlap5PrimeGeneGrps

```
public java.util.ArrayList getOverlap5PrimeGeneGrps()
```

---


### getRank

```
public int getRank()
```

---


### getStart

```
public int getStart()
```

---


### getEnd

```
public int getEnd()
```

---


### getNeighborhood

```
public int getNeighborhood()
```

---


### setNeighborhood

```
public void setNeighborhood(int neighborhood)
```

---


### getNeighboringGeneGrps

```
public java.util.ArrayList getNeighboringGeneGrps()
```

---


### getScore

```
public double getScore()
```

---


### getClosestBindingRegion

```
public BindingRegion getClosestBindingRegion()
```

---


### setClosestBindingRegion

```
public void setClosestBindingRegion(BindingRegion closestBindingRegion)
```

---


### getLength

```
public int getLength()
```

:   End-Start+1

---


### getGcContent

```
public double getGcContent()
```

---


### setGcContent

```
public void setGcContent(double gcContent)
```

---


### setChromosome

```
public void setChromosome(java.lang.String chromosome)
```


---


|  |  |  |
| --- | --- | --- |
| **Overview** \| **Package** \| **Class** \| **Use** \| **Tree** \| **Deprecated** \| **Index** \| **Help** | |  |
| **PREV CLASS**   **NEXT CLASS** | **FRAMES**    **NO FRAMES**     **All Classes** |  |
| SUMMARY: NESTED \| FIELD \| CONSTR \| METHOD | DETAIL: FIELD \| CONSTR \| METHOD |  |


---
